# Marketing and Pricing Strategies of Blockbuster Drugs in the South Korean Market: A 15-Year Retrospective Cohort Study for Choline Alfoscerate

**DOI:** 10.3389/fphar.2020.00232

**Published:** 2020-03-06

**Authors:** Jeewon Park, SeungJin Bae, Tae-Jin Lee, Kyung-Bok Son

**Affiliations:** ^1^College of Pharmacy, Ewha Womans University, Seoul, South Korea; ^2^Institute of Health and Environment, Seoul National University, Seoul, South Korea; ^3^Department of Public Health Science, Graduate School of Public Health, Seoul National University, Seoul, South Korea

**Keywords:** competition, marketing and pricing strategies, pharmaceuticals, pharmaceutical expenditure, South Korea

## Abstract

**Introduction:**

Understanding marketing strategies and price competition among manufacturers is essential to manage health care expenditures, particularly those related to blockbuster drugs.

**Objectives:**

To assess marketing and pricing strategies of blockbuster drugs in South Korea.

**Methods:**

Baseline information on manufacturers who were granted marketing approval for choline alfoscerate in various forms was retrieved. Accumulation of manufacturers in the market was also identified, and manufacturers were categorized into first movers and latecomers based on their marketing time. Then, an event history analysis and a regression analysis were applied to estimate the duration of marketing and their price competition.

**Results:**

Currently, 109, 83, and 26 manufacturers produce choline alfoscerate in capsule, tablet, or syrup form, respectively, indicating that many manufacturers have marketed generics and the majority of the generics are categorized as latecomers. The size of the manufacturer was a significant factor in marketing new medicines, while the variable was not related to the marketing of modified drugs. Furthermore, price competition in the market was rare and only a few major firms initiated price competition.

**Conclusion:**

The Korean market appears to be an example of perfect competition when we focus on the number of manufacturers. However, the market is near-monopolistic when examining the price of generic drugs. While product competition between different forms of drugs is effective in lowering price, product competition within the same form of a drug does not exist in the market.

## Introduction

Competition in the health sector is an important and contentious issue (Fuchs, 1988; Gaynor and Vogt, 2000), and pharmaceuticals are no exception with the patent system being closely related to their competition in the market (Kanavos et al., 2007; Garattini and Padula, 2018). The World Trade Organization (WTO) requires all countries to adopt a 20-year patent system as a condition of membership in the WTO (Hestermeyer, 2007; Athreye et al., 2009; Son and Lee, 2018), and accordingly, the member countries have established an appropriate patent system, a factor that may undermine the availability and affordability of generic drugs (generics) (Oliveira et al., 2004; Chaves and Oliveira, 2007; Luo et al., 2014). Thus, a traditional approach to examining the intrinsic characteristics of market failure with respect to pharmaceuticals has been adopted when analyzing competition in the pharmaceutical literature, with a particular focus on price competition immediately following patent expiration and the subsequent introduction of generics (Cook, 1998; Lexchin, 2004; Clarke and Fitzgerald, 2010; Lexchin, 2017; Beall et al., 2018).

Containing health expenditure has been a policy priority in high-income countries (Jakovljevic and Ogura, 2016; Dieleman et al., 2017) as well as low- and middle-income countries (Rancic and Jakovljevic, 2016; Jakovljevic et al., 2017). In pharmaceutical sector, generic entry is a key driver of competition and cost containment measures. The entry of generics will trigger price competition and alter the market structure significantly. Nonetheless, competition issues regarding generics have been realized in recent years. First, the rising prices of generics due to monopolies have been continuously reported in high-income countries such as the United States where tighter pricing and reimbursement schemes do not exist (Schweitzer, 2013; Kesselheim et al., 2015; Worth, 2015; Dave et al., 2017b). Thus, concerns over rising drug prices for generics have gathered special attention from patients, physicians, and policy makers in the United States. For instance, the price of digoxin, which was approved by the Food and Drug Administration (FDA) in 1954, increased by 637% in a single year (Kesselheim et al., 2015). Second, a lack of price competition among a number of generics has been reported in other high-income countries, particularly in South Korea where many manufacturers have marketed generics (Bae, 2019; Son, 2019). For instance, 119 manufacturers have marketed atorvastatin 10 mg in the South Korean market, implying that the pharmaceutical market is an example of highly competitive market in which many manufacturers produce fully homogeneous generics for the market (Son, 2019).

This study is interested in the lack of price competition among generics, which is another type of market failure for pharmaceuticals. It is well documented that incentives to lower maximum allowance prices of generics do not exist in the South Korean market (Bae, 2019). Furthermore, generic latecomers, i.e., generics that have been granted marketing authorization after the market has been saturated with previously marketed generics, account for a large portion of the manufacturers who continue to enter the market without price competition or discounted prices (Son, 2019). However, no study has empirically examined the price competition among generic manufacturers in South Korea. Because understanding marketing strategies and price competition among manufacturers is essential to manage health care expenditures (Belloni et al., 2016; Angelis et al., 2017), particularly those related to blockbuster drugs, it is necessary to identify policy options that address sustainability issues from the perspectives of health financing.

In this context, this study has two aims. First, we trace the manufacturers of generics who have entered the market, sort the manufacturers into first movers and latecomers, analyze their accumulation in the market, calculate the duration between the date of marketing approval for the first drug and the remaining generics, and then perform an event history analysis to determine a statistical estimation of duration. Second, we retrieve price information with respect to the identified drug and apply a regression model to assess price competition among manufacturers.

To this end, we selected choline alfoscerate as the subject of this study. Choline alfoscerate is a semisynthetic derivative of phosphatidylcholine, a drug that has been determined to improve cognitive deficit and facilitate learning and memory for patients with Alzheimer’s disease (Moreno, 2003). In South Korea, choline alfoscerate is prescribed for patients with secondary symptoms of and degenerative changes from cerebrovascular defects or degenerative organic brain syndrome and for patients with pseudo-depression. The patients are reimbursed for the cost of the drug under the National Health Insurance Service (NHIS) when presenting with the aforementioned indications. Thus, the prescription for choline alfoscerate and the corresponding pharmaceutical expenditures in the market have continuously increased. For instance, the NHIS claimed 113.6 billion Korean won (KRW, approximately 96 million US Dollar) for choline alfoscerate in the first half of 2019, an amount that reflected an increase of 18% compared to the previous semiannual report (Yang, 2019).

## Materials and Methods

### Study Subjects

We are interested in the market competition of manufacturers who were granted marketing approval for choline alfoscerate in various forms by the Ministry of Food and Drug Safety (MFDS) in South Korea from 2005 to 2019. Choline alfoscerate is currently available on the market in three forms, capsule, tablet, and syrup. Thus, all three forms of choline alfoscerate are included in this study.

### Data Source

Similar to the FDA, the MFDS constructs a publicly available dataset that provides information regarding approved drugs, including the generic and proprietary name of the drug, the date of marketing approval, and the manufacturers of the drug^1^. We selected drugs containing choline alfoscerate 400 mg as the generic name of the drug and then collected information regarding their unit price and manufacturers.

Prices were retrieved based on the information provided by the Health Insurance Review and Assessment Service^2^ (HIRA). Using this source allowed us to observe price changes of certain drugs. Information regarding manufacturers was obtained from two sources. First, to collect information on manufacturers and understand the financial resources of the companies that conduct external audits, we used KISVALUE, an analytical dataset. Using these data, we categorized the manufacturers into major-, medium-, and small-sized manufacturers. Second, we retrieved documents from the Ministry of Health and Welfare website to identify manufacturers designated as innovative manufacturers^3^. In this study, we used the variable “innovative manufacturer” as a proxy for the capabilities of manufacturers in the areas of research and development (Ministry of Health and Welfare, 2012; Son, 2018).

### Statistical Analysis

This study addresses manufacturers who produce and market choline alfoscerate in South Korea. First, we used descriptive analysis to capture the characteristics of the choline alfoscerate market, including the number of manufacturers, their accumulation in the market, and the price sorted by drug form, i.e., capsule, tablet, or syrup. We categorized manufacturers into first movers and latecomers. First movers were defined as manufacturers who entered the market within two and half years after the date of the first entry, while latecomers were defined as manufacturers who entered the market after two and half years (Son, 2019). The market penetration of generics in the South Korean market reached saturation after two and a half years from the date that the first generic drug entered the market (Yang et al., 2017). Then, a Chi-square test was applied to analyze differences in the variables for financial resources and designation as innovative manufacturers between the two groups, i.e., first movers and latecomers.

Second, we applied two inferential statistics to estimate the marketing of drugs and their price competition, an event history analysis and a regression analysis. In the event history analysis, we applied a proportional hazard model to estimate the impact of the characteristics of manufacturers on the duration. In our model, we included two categorical variables. Specifically, we used the size of the manufacturers based on financial resources and on the designation of being an innovative manufacturer as the variables of interest in this model. We also conducted a regression analysis to understand price competition with respect to choline alfoscerate. Variables of interest, such as the formulation of drugs and the size of the manufacturers, were added to the model to elucidate factors that affect the price of the drug at a certain point in time. We used heteroscedastic-consistent standard errors for the analysis to obtain a correct standard error. Data management and analysis were performed using R statistical software (version 3.4.3). Statistical significance is denoted by *p*-values less than 0.05.

## Results

### Characteristics of Reference Products

Table 1 provides information on the characteristics of the eligible products for this study. Currently, choline alfoscerate 400 mg is available on the market in three forms, capsule; tablet; and syrup. Choline alfoscerate was available on the market in capsule form on 26 October 2005, but not granted marketing authorization in tablet or syrup form until 2013 and 2019, respectively. As of October 2019, in the South Korean market, 109, 83, and 26 manufacturers produce choline alfoscerate 400 mg in capsule, tablet, or syrup form, respectively.

**TABLE 1:** Characteristics of reference products.

**Portfolio**	**Product characteristics**	**Date of the first entry**	**Number of manufacturers**
Capsule	400 mg choline alfoscerate	10/26/2005	109
Tablet	400 mg choline alfoscerate	8/21/2013	83
Syrup	400 mg choline alfoscerate	3/29/2019	26

### Products in the Market

#### Cumulative Number of Choline Alfoscerate

**FIGURE 1:**
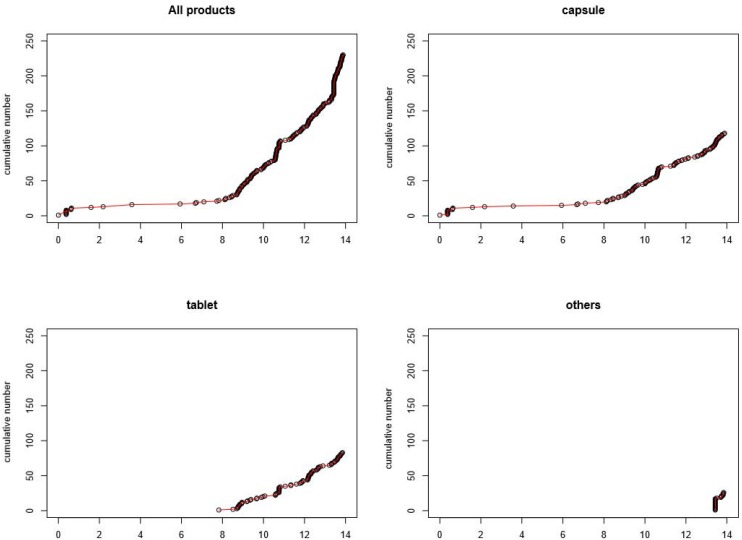
Cumulative number of choline alfoscerate sorted by various formulations. Note *X*-axis indicates duration in years starting at the date of first entry of choline alfoscerate.

Figure 1 presents an overview of the cumulative number of available choline alfoscerate drugs sorted by form. The first graph in Figure 1 presents a cumulative curve of the three forms of choline alfoscerate. As presented in Table 1, choline alfoscerate in capsule form was granted market authorization on 26 October 2005 (duration 0), and additional 10 drugs in capsule form were approved by the MFDS in 2006 (duration 1). Drugs in tablet form were available on the market on 21 August 2013 (duration 8). Finally, in October 2019, 218 choline alfoscerate drugs were available on the market. The remaining graphs in Figure 1 present curves for the capsule, tablet, and syrup forms of choline alfoscerate. The syrup form of choline alfoscerate was authorized on 29 March 2019 (duration 14).

#### Two Types of Generics

Table 2 displays the characteristics of the first movers and the latecomers of the various forms of choline alfoscerate. Specifically, 13 products (12%) of the drugs in capsule form were categorized as first movers, while 96 products (88%) were categorized as latecomers. Additionally, we sorted manufacturers who produced drugs based on their financial resources and their designation as innovative manufacturers. Approximately, 24, 6, and 5% of manufacturers from among the major-, medium-, and small-sized categories, respectively, were defined as first movers of choline alfoscerate in capsule form. Similarly, 30 and 7% of manufacturers from among innovative manufacturers and non-innovative manufacturers, respectively, were categorized as first movers of the drugs.

**TABLE 2:** Characteristics of the first movers and latecomers of the various forms of choline alfoscerate.

	**Capsule (*n* = 109)**			**Tablet (*n* = 83)**			**Syrup *N* = 26**
	**First mover *N* = 13**	**Late comers *N* = 96**	***P*-value**	**First mover *N* = 21**	**Late comers *N* = 62**	***P*-value**	
**Size**							
Major	9	29	0.0351	12	15	0.0203	12
Medium	3	49		7	38		14
Small	1	18		2	9		0
**Innovative manufacturers**							
Yes	7	16	0.0065	7	10	0.1689	9
No	6	80		14	52		17

The same information regarding the tablet and syrup forms of choline alfoscerate is calculated. Specifically, we found the 25% of the manufacturers were categorized as first movers of choline alfoscerate in tablet form and that approximately 44, 16, and 18% of manufacturers among the major-, medium-, and small-sized manufacturers, respectively, were categorized as first movers of choline alfoscerate in tablet form. Similarly, 41 and 21% of manufacturers among innovative manufacturers and non-innovative manufacturers, respectively, were categorized as first movers of the drugs. Drugs in syrup form could not be categorized into first movers and latecomers due to their more recent marketing, which occurred on 29 March 2019.

#### Cox Proportional Hazards Model

Table 3 provides interpretations regarding the size of the manufacturer based on its financial resources and the designation as an innovative manufacturer on the duration using the Cox proportional hazards model. The duration was defined as the year difference between the date of the first drug entry and that of the remaining drugs. A negative coefficient implies a long time to market entry, while a positive coefficient indicates a prompt marketing of the drug. It is noted that the time to market authorization of capsule forms for small-sized manufacturers was delayed compared to the time for major-sized manufacturers. However, the remaining variables for size and innovation were not significantly delayed nor did they prompt the marketing of choline alfoscerate in capsule form. The same model was applied for tablet forms of choline alfoscerate. None of the variables were significantly associated with the time for marketing authorization of choline alfoscerate in tablet form.

**TABLE 3:** Results from the Cox model with duration starting at the date of the first drug entry.

	**Capsule (*n* = 109)**			**Tablet (*n* = 83)**		
	**Coefficient**	**Standard error**	***P*-value**	**Coefficient**	**Standard error**	***P*-value**
**Size (reference major)**						
Medium	−0.5289	0.2750	0.0544	−0.4701	0.2957	0.1120
Small	−0.8117	0.3373	0.0161	−0.6612	0.4022	0.1000
**Innovative manufacturers (reference no)**						
Yes	0.1676	0.3086	0.5870	−0.3679	0.3312	0.2670

### Price Competition in the Market

Table 4 presents the information on the unit price of choline alfoscerate sorted by form and manufacturer as of October 2019. The table indicates that the maximum allowance price for choline alfoscerate under the NHIS is currently 523 KRW (approximately 0.44 USD). Specifically, the average price for choline alfoscerate is 519.2 KRW in capsule form, 520.9 KRW in tablet form, and 522.3 in syrup form. However, the median price of those medicines was 523 KRW, i.e., the maximum allowance price. Similarly, the average prices of choline alfoscerate in capsule form were 515.5, 520.4, and 520.5 KRW for major-, medium-, and small-sized firms, respectively, and the median price of the medicines was also 523 KRW, indicating that price competition among manufacturers is rare in the South Korean market.

**TABLE 4:** Information on the unit price of choline alfoscerate sorted by form and manufacturers as of October 2019.

	**Capsule**				**Tablet**				**Syrup**		
	**All**	**Major**	**Medium**	**Small**	**All**	**Major**	**Medium**	**Small**	**All**	**Major**	**Medium**
Maximum	523.0	523.0	523.0	523.0	523.0	523.0	523.0	523.0	523.0	523.0	523.0
Third quartile	523.0	523.0	523.0	523.0	523.0	523.0	523.0	523.0	523.0	523.0	523.0
Median	523.0	523.0	523.0	523.0	523.0	523.0	523.0	523.0	523.0	523.0	523.0
First quartile	523.0	508.5	523.0	523.0	523.0	517.5	523.0	523.0	523.0	523.0	523.0
Minimum	480.0	480.0	480.0	482.0	480.0	496.0	480.0	521.0	508.0	508.0	522.0
Mean	519.2	515.5	520.4	520.5	520.9	518.7	521.9	522.8	522.3	521.7	522.9
Standard deviation	10.55	11.97	9.32	9.92	6.63	7.51	6.63	0.60	2.93	4.31	0.26

*523 Korean Won is 0.44 US Dollar.*

Table 5 presents the results of a regression model on the price of eligible drugs. Model 1 regressed the price based on two variables, the formulation of the drug and the size of the manufacturer based on financial resources. Not surprisingly, the newly approved formulation (syrup form) was more expensive than the reference drugs, i.e., the capsule form. Furthermore, the size of the manufacturer, i.e., medium- and small-sized was closely related with the expensive price of the drug compared to the reference drug, i.e., major manufacturer. In models 2 and 3, we separated the formulations and then applied a simple regression. Models 2 and 3 regressed the price on the size of the manufacturer for capsule and tablet forms, respectively. In model 2, the size of the manufacturer was not significantly related to the price of the drug in capsule form. However, the same variable (particularly, small-sized manufacturer) was significantly related to the price of the drug in tablet form. It is further noted that the capsule and tablet forms were granted marketing authorization in different years. Specifically, the drug in capsule form has been available since 2005, while the first choline alfoscerate in tablet form was not approved until 2013. To adjust for the time difference, we applied model 2-1 to the capsule form of choline alfoscerate. For this model, we retrieved price information for the capsule form as of December 2016. Thus, medicines in capsule form that were granted marketing authorization after December 2016 were omitted in this model. It was determined that the variables for size, i.e., small- and medium-sized, were significantly related to the price of the drug in capsule form in 2016.

**TABLE 5:** Factors affecting the price of choline alfoscerate.

	**Model 1 (*N* = 206) All forms, as of 2019**			**Model 2 (*N* = 100) Capsules only, as of 2019**			**Model 2_1 (*N* = 59) Capsules only, as of 2016**			**Model 3 (*N* = 80) Tablets only, as of 2019**		
	**Estimate**	**Std. error**	***P*-value**	**Estimate**	**Std. error**	***P*-value**	**Estimate**	**Std. error**	***P*-value**	**Estimate**	**Std. error**	***P*-value**
**Formulation (reference capsule)**												
Tablet	1.768	1.2166	0.1477									
Syrup	3.638	1.1843	0.0024									
**Size (reference major)**												
Medium	3.105	1.2553	0.0142	3.693	2.2366	0.1019	5.1012	2.0538	0.0160	3.116	1.8017	0.0876
Small	3.663	1.8245	0.0460	3.735	3.0571	0.2247	5.0655	2.0580	0.0169	4.077	1.4865	0.0075

## Discussion

### Summary of Findings

This study presents several interesting findings. First, we reaffirmed that many manufacturers have marketed generics in the South Korean market and that the majority of the generics are categorized as latecomers. For example, while 218 choline alfoscerate drugs are currently available on the market, 88% of the choline alfoscerate drugs in capsule form are categorized as latecomers. Second, manufacturers exhibit different behaviors when marketing the various forms of the drug. For example, choline alfoscerate in capsule form is a new drug in the market, whereas the drug in tablet form is a modified drug that improves the method of storage and the method of taking the drug. Furthermore, we captured different results in the marketing of new and modified drugs in our event history analysis. For example, we found that the size of the manufacturer was a significant factor in marketing new medicines, while this variable was not related to the marketing of modified drugs. Finally, by analyzing price competition among manufacturers in the South Korean market, we confirmed that price competition in the market was rare and that only a few major firms initiated weak price competition.

### Extraordinary Pharmaceutical Market in South Korea

Son (2019) provides an overview of the cumulative number of available generics on the South Korean market for atorvastatin and rosuvastatin, both of which are well-known blockbuster drugs (Son, 2019). This study concluded that the number of generics for atorvastatin and rosuvastatin increased sharply after the first generic drug entered the market, remained unchanged for a certain period time, and then increased again at a certain point. It was determined that 119 and 116 manufacturers in the South Korean market produce atorvastatin 10 mg and rosuvastatin 10 mg, respectively. We confirmed similar market trends for the various forms of choline alfoscerate, another well-known blockbuster drug on the South Korean market. Accordingly, the results allow us to conclude that the cumulative number of generics on the South Korean market is extraordinary when compared to other high-income countries, such as the United States, Germany, Canada, and Japan, where fewer than 20 generic drugs are paired with the original drugs (Fischer and Stargardt, 2016; Luo et al., 2016; Zhang et al., 2016).

The classic economic theory of perfect competition assumes a market in which products are fully homogeneous, consumers are price takers, and no entry or exit barriers exist for the many manufacturers (McNulty, 1967; Kesselheim et al., 2015). Furthermore, it has been well documented that increases in generic drug prices are strongly associated with market competition, implying that an increase in competition causes a decrease in drug prices (Dave et al., 2017a, b). In other words, manufacturers that produce generic drugs in less competitive markets have greater flexibility with respect to increasing drug prices. For example, manufacturers with multiple drug portfolios may increase drug prices in non-competitive markets to complement lower profits (or drug prices) in competitive markets.

We observed contrasting trends in the South Korean market, a market that appears to be an example of perfect competition when we focus on the number of manufacturers. However, the market is more monopolistic or near-monopolistic when examining the price of generic drugs. The price of the majority of generic drugs adheres to the maximum allowance price. While some may argue that the maximum allowance price is a marginal cost in a perfect competitive market, the price of drugs in South Korea, particularly the price for generic drugs, is higher than the price in other high-income countries (Kim et al., 2010). Therefore, many manufacturers of generic drugs, as well as latecomers, steadily enter the market even after the market has been saturated with a number of generic drugs that are expected to produce profits based on their price.

This gives rise to the question, “What is the role of latecomers in the South Korean market?” Product competition based on quality differentiation provides some clues to understanding the market. In a market with product competition, manufacturers attempt to differentiate their products and become more competitive based on their quality rather than on their price (Cheng and Peng, 2014). In other words, manufacturers differentiate their products to avoid price competition and continue to set higher prices for longer periods to maximize profits.

In this study, we confirmed the existence of product competition in the South Korean market, and as such, the various manufacturers who produce choline alfoscerate differentiate their products. For example, choline alfoscerate was first available on the market in 2005 in capsule form. However, because drugs in capsule form are vulnerable to humidity and high temperatures, they are press packaged, which is less convenient for distribution and storage than bottle packaging (Choi and Cho, 2013). Furthermore, the liquid components contained in a soft capsule are absorbed into the water-soluble gelatin coating, a factor that may lower the effectiveness and impact the safety of the drug due to microbial alteration (Choi and Cho, 2013). To address these issues, choline alfoscerate in tablet form was marketed in 2013, and the drug in syrup form, which was designed for geriatric patients who had problems swallowing, was made available in 2019.

A regression model revealed a consistent result regarding product competition and its effects on price. While choline alfoscerate in syrup form, which is a newly modified drug, was significantly more expensive than the drug in capsule form, the drug in tablet form was not significantly more expensive than the drug in capsule form. The difference between the drug in syrup form and the drug in tablet form could be explained by the characteristics of the drug itself. The drug in tablet form, which was marketed in 2013, is no longer considered a newly modified drug on the market.

However, product competition does not fully explain the South Korean market. More specifically, while product competition between different forms of drugs is effective in lowering drug price, product competition within the same form of a drug does not exist in the market. As previously explained, price competition within the same form is rare in the South Korean market, and unless policies are established to introduce competition in response to these issues, we may continue to see cases of a number of high-priced generic drugs in the market.

Policies to expand generic penetration could be enacted to address the issue (Iijima et al., 2004; Iizuka and Kubo, 2011; Jakovljevic et al., 2014). We demonstrated that latecomers steadily enter the market even after the market has been saturated by a number of generic drugs with expected profits that are mainly the result of the price of the drugs. Furthermore, generic penetration in the market is marginal, with approximately 39.3% of this penetration being based on volume (Yang et al., 2017), when compared to other high-income countries (Saha et al., 2006; Berndt and Aitken, 2011; Dave et al., 2017b). Thus, policies to expand the volume of generic drugs could be established to introduce competition among generic drugs. If so, manufacturers of generic drugs, particularly major manufacturers (as determined by financial resources) could reasonably anticipate profits based on the volume of drugs rather than on the price of drugs and thereby more actively initiate price competition in the market.

Second, entry barriers or exit strategies would be created to manage the number of generic drugs. Currently, coorganized bioequivalence tests (coorganized tests) among generic manufacturers and original equipment manufacturers (OEMs) have been authorized in the marketing of generic drugs since November 2011 (Korea Food and Drug Administration, 2011). Thus, excessive number of manufacturers exist in the market, most of whom are small- and medium-sized manufacturers without capability in research and development (Proffitt, 2013; Korea Health Industry Development Institute, 2017). The South Korean government is set to abolish the regulations on coorganized tests in 2023 due to expectation of decreased number of generic manufacturers (Ministry of Health and Welfare, 2019).

Third, to consolidate the market power or intervene in market with a number of manufactures, merging with other manufacturers is a recommendation worthy of consideration (The Lancet, 2015; IQVIA, 2019). For instance, Teva, the world’s largest generic manufacturer, recently acquired Allergan, the third largest manufacturer. In this same vein, the Japanese government suggested merging with other manufacturers to achieve economies of scale in production and research in the pharmaceutical sector where a number of medium- and small-sized manufactures exist (Ministry of Health Labour and Welfare, 2015; IQVIA, 2019). It is noteworthy that monopolies or duopolies in the pharmaceutical market could lead to increased drug prices, a reality that is frequently reported in the United States market (Schweitzer, 2013; Kesselheim et al., 2015; Worth, 2015; Dave et al., 2017b). However, consolidated market power is currently required to address rare competition among excessive number of generic manufacturers in the South Korean market (Proffitt, 2013). In a consolidated market, manufacturers could expect profits that are mainly the result of the quantity of the drugs instead of the price of the drugs, which demonstrates the current competition in South Korea.

### Limitations

This study has some limitations. First, we selected choline alfoscerate to understand market dynamics in South Korea, indicating that findings from this study may not be generalizable to other drugs. However, similar trends were observed in other studies (Son, 2019). Second, we used drug approval data and price information provided by the MFDS and HIRA, respectively. However, we could not include information of drug utilization. More specifically, the market share of each drug could not be assessed in this study.

## Conclusion

Many manufacturers have marketed generics in the South Korean market and that the majority of the generics are categorized as latecomers. Thus, the cumulative number of generics on the South Korean market is extraordinary when compared to other high-income countries, where fewer than 20 generic drugs are paired with the original. The Korean market appears to be an example of perfect competition when we focus on the number of manufacturers. However, the market is near-monopolistic when examining the price of generic drugs. The price of the majority of generic drugs adheres to the maximum allowance price. While product competition between different forms of drugs is effective in lowering price, product competition within the same form of drugs does not exist in the market. Policies to expand generic penetration, to establish entry barriers, and to consolidate the market power could be enacted to address the issue.

## Data Availability Statement

Publicly available datasets were analyzed in this study. These data can be found here: https://nedrug.mfds.go. kr/pbp/CCBGA01/getItem?infoName=%ED%97%88%EA%B0 %80&totalPages=4&limit=10&searchYn=true&page=1&&open DataInfoSeq=7, https://biz.hira.or.kr/popup.ndo?formname=qya_bizcom%3A%3AInfoBank.xfdl&framename=InfoBank.

## Author Contributions

JP and K-BS developed the concept of the manuscript and undertook the analysis. All the authors were involved with developing and refining the manuscript and approved the final draft before submission.

## Conflict of Interest

The authors declare that the research was conducted in the absence of any commercial or financial relationships that could be construed as a potential conflict of interest.

## Footnotes

1 https://nedrug.mfds.go.kr/pbp/CCBGA01/getItem?infoName=%ED%97%88%EA%B0%80&totalPages=4&limit=10&searchYn=true&page=1&&openDataInfoSeq=7

2 https://biz.hira.or.kr/popup.ndo?formname=qya_bizcom%3A%3AInfoBank.xfdl&framename=InfoBank

3 http://www.mohw.go.kr/react/al/sal0301vw.jsp?PAR_MENU_ID=04&MENU_ID=0403&page=1&CONT_SEQ=347199&SEARCHKEY=TITLE&SEARCHVALUE=%ED%98%81%EC%8B%A0%ED%98%95+%EC%A0%9C%EC%95%BD%EA%B8%B0%EC%97%85
